# 8-Chloroadenosine Sensitivity in Renal Cell Carcinoma Is Associated with AMPK Activation and mTOR Pathway Inhibition

**DOI:** 10.1371/journal.pone.0135962

**Published:** 2015-08-27

**Authors:** Alper Y. Kearney, You-Hong Fan, Uma Giri, Babita Saigal, Varsha Gandhi, John V. Heymach, Amado J. Zurita

**Affiliations:** 1 Department of Genitourinary Medical Oncology, Division of Cancer Medicine, The University of Texas MD Anderson Cancer Center, Houston, Texas, United States of America; 2 Department of Thoracic & Head and Neck Medical Oncology, Division of Cancer Medicine, The University of Texas MD Anderson Cancer Center, Houston, Texas, United States of America; 3 Department of Experimental Therapeutics, The University of Texas MD Anderson Cancer Center, Houston, Texas, United States of America; Université catholique de Louvain, BELGIUM

## Abstract

The adenosine analog 8-chloroadenosine has been shown to deplete ATP and inhibit tumor growth in hematological malignancies as well as in lung and breast cancer cell lines. We investigated effects of 8-chloroadenosine on clear cell (cc) renal cell carcinoma (RCC) cell lines. 8-chloroadenosine was effective against ccRCC cell viability in vitro, with IC_50_ ranging from 2 μM in the most sensitive CAKI-1 to 36 μM in the most resistant RXF-393. Proteomic analysis by reverse-phase protein array revealed that 8-chloroadenosine treatment leads to inhibition of the mTOR pathway. In time-course experiments, 8-chloroadenosine treatment rapidly activated AMPK, measured by AMPK and ACC phosphorylation, and subsequently caused dephosphorylation of p70S6K and ribosomal protein RPS6 in the sensitive cell lines. However, in the resistant cell lines, AMPK activity and the mTOR pathway were unaffected by the treatment. We also noted that the resistant cell lines had elevated basal levels of phospho RPS6 and AKT. Inhibition of PI3K pathway enhanced the efficacy of 8-chloroadenosine across all cell lines. Our observations indicate that 8-chloroadenosine activity is associated with inhibition of the mTOR pathway, and that phospho RPS6 and PI3K pathway activation status may determine resistance. Among solid tumors, RCC is one of the few susceptible to mTOR inhibition. We thus infer that 8-chloroadenosine may be effective in RCC by activating AMPK and inhibiting the mTOR pathway.

## Introduction

The adenosine analog 8-chloroadenosine is a novel anticancer drug candidate. In vitro, it is potently cytotoxic against cancer cell lines from different tumor types, including leukemia [1], mantle cell lymphoma [2], multiple myeloma [3–5], breast [6], and lung cancers [7]. It is also cytotoxic in combination with TRAIL (TNF-related apoptosis inducing ligand) in hepatocarcinoma [8]. In vivo, 8-chloroadenosine inhibits growth of breast cancer [9], hepatocarcinoma, and leukemia in mice [10]. A phase I clinical trial of intravenous 8-chloroadenosine in chronic lymphocytic leukemia patients is in progress [11].

Treatment with 8-chloroadenosine in vitro results in induction of cell cycle arrest and apoptosis [7, 12]; however, the molecular basis for such effects is not fully understood. 8-chloroadenosine, once in the cell, is converted to its triphosphate form, 8-chloro-ATP, through ATP synthase, thus depleting intracellular ATP [13]. In mantle cell lymphoma cell lines, 30–60% ATP depletion was achieved after 24 hours treatment, and 8-chloro-ATP accumulation was highly associated with cell death [2]. Different groups have reported diverse consequences of 8-chloroadenosine treatment in various cancer types. In multiple myeloma cells, it is preferentially incorporated into mRNA, and at a lesser rate into tRNA and rRNA, resulting in inhibition of RNA synthesis in 4 hours. It has not been set clear whether 8-chloroadenosine metabolites similarly inhibit DNA synthesis [2, 3]. In myelocytic leukemia cells, it inhibited topoisomerase II and induced DNA double-strand breaks. In lung cancer cells, 8-chloroadenosine interfered with actin polymerization [14], upregulated p14ARF through E2F1 [7], and increased DNA damage and chromosome missegregation [15]. Finally, in breast cancer cells, it depleted cyclin E, inhibited the mammalian target of rapamycin (mTOR) pathway, and induced autophagy [2, 6]. To our knowledge, 8-chloroadenosine has not been studied in renal cell carcinoma (RCC).

RCC is a common malignancy, with >65,000 patients diagnosed annually in the US. The clear cell (ccRCC) is the most frequent RCC type. There are limited established treatment options for ccRCC patients, including surgical resection, VEGF pathway inhibitors, and high-dose interleukin-2 (IL-2), although novel immunotherapies will likely join this list soon. The rapalogs everolimus and temsirolimus are also approved and in clinical use for advanced ccRCC. Indeed, a limited number of ccRCC tumors are profoundly sensitive to mTOR inhibition therapy, but a larger proportion, >25%, is refractory [16]. Moreover, in those that are initially sensitive to mTOR inhibition, resistance develops typically after weeks or a few months. mTOR inhibitor resistance is a major cause of therapy failure in ccRCC. In order to overcome this problem, new generation mTOR inhibitors and combination therapies of rapalogs and other drugs, including PI3K and AKT inhibitors, are being developed. Novel treatments are needed to expand options for RCC patients beyond the few currently available targeted drugs.

Here, we present our findings on the effects of the novel candidate drug, 8-chloroadenosine, in ccRCC. We show that 8-chloroadenosine is effective on ccRCC cells lines and leads to AMPK activation and mTOR pathway inhibition. Furthermore, we provide evidence that PI3K pathway activation is associated with resistance to 8-chloroadenosine, and that PI3K inhibition synergizes with 8-chloroadenosine treatment.

## Materials and Methods

### Cell culture and cell cycle assay

Human ccRCC cell lines A498, ACHN, CAKI1, RXF393, SN12C, TK10, and UO31 were obtained from the National Cancer Institute (NCI60 collection); 786-O cell line was obtained from American Type Culture Collection (ATCC), and RCC4 cell line [17] was a kind gift from Dr. Eric Jonasch (MD Anderson Cancer Center [MDACC], Houston, TX). Cells were grown in RPMI 1640 with 10% FBS, 1% 100x L-glutamine supplement, 1% 100x penicillin/streptomycin, and 1% 100x non-essential amino acid supplement at 37°C and 5% CO_2_. Bright field images were taken at 10x and 40x magnification.

To assess phases of cell cycle, cells treated with 8-chloroadenosine (Tocris Bioscience, UK) or vehicle and incubated for 48 hours. The drug was added only once at the beginning of the experiment. The cells were washed with PBS, fixed in cold 70% ethanol, washed again with PBS, treated with RNase and stained with 50 μg/ml propidium iodide solution. Cell cycle data was collected on FACS Canto II flow cytometer (BD Biosciences, USA) and analyzed with FlowJo software (FlowJo LLC, USA). Two-tailed paired t-test was used to obtain p-values.

### Proliferation assay

Cells were plated in 96-well plates and treated once with vehicle, 8-chloroadenosine (Tocris Bioscience, UK), the PI3K p110α inhibitor A66 (Selleck Chemicals, USA), or 1:1 combination of the latter two. Cell TiterGlo (Promega, USA) and Optima plate reader were used for assessing cell viability after 96 hours, and the Calcusyn software (Biosoft, UK) for calculating IC_50_ and synergy (CI; combination index) values. Synergy between 8-chloroadenosine and A66 was measured by Chou-Talalay method in the Calcusyn software.

### ATP quantification assay

Cells were plated in 96-well plates and treated once with vehicle or 40 μM 8-chloroadenosine. ATP levels in the cells were measured after 6 hours using CellTiter Luminescent assay (Promega, USA) per instructions of the manufacturer. ATP levels in each cell line treated with vehicle vs. 8-Cl-adenosine and ATP levels between sensitive and resistant cell lines were compared by two-tailed t-test.

### Reverse-phase protein assay

Cells were grown in RPMI, treated once with 40 μM 8-chloroadenosine or vehicle for 24 hours. Cells were washed with cold PBS prior to lysis, and lysates were prepared on ice. Protein concentration was quantified with the DC Protein Assay (Bio-Rad, USA). RPPA was performed as previously described [18]. The RPPA data was median normalized between samples, log2 transformed, and filtered for >1.5 fold difference. Coefficients were calculated using Pearson correlation. P-values were obtained using two-tailed paired t-tests for changes in phosphorylated S6 levels. Heatmaps were drawn using Java TreeView v1.1.6.r2 software (Alok Saldanha, Sourceforge.net, USA).

### Western blot

Cells were lysed in RPPA lysis buffer supplemented with NaF, NaVO4, PMSF, protease inhibitor tablet complete (Roche, USA), phosphatase inhibitor tablet PhosSTOP (Roche, USA). Lysates were mixed with sample buffer supplemented with beta-mercaptoethanol and boiled on heatblock for 5 minutes and then cooled on ice prior to running at 70V-120V on 10% polyacrylamide or 5%-18% gradient (Bio-Rad, USA) gels. Proteins were wet-transferred to nitrocellulose membrane, and probed with primary and HRP-conjugated secondary antibodies in 5% skim milk in TBS-T, and signal was developed using substrate solution (ECL Western and Pico kits, Life Technologies, USA). Antibodies against phospho S6 S235/236 (2211), AMPKα T172 (2532), ACC S79 (3661), p70 S6K T389 (9205), AKT S473 (9271), ERK T202/204 (9101), GSK3α.S21/GSK3β.S9, and antibodies against total S6 (2217), AKT (9272), GSK3α/β (SC7291), beta catenin (9562), vinculin (V9131), and beta actin were purchased from Cell Signaling Technology (USA), Santa Cruz Biotechnology (USA), and Sigma-Aldrich (USA). Western blot bands were quantified for total intensity using ImageJ software v1.48 (imagej.nih.gov/ij, NIH, USA). Band intensities were corrected for background and loading control. For each blot, the values were standardized with the highest value set to 100.

## Results

### 8-chloroadenosine is cytotoxic in ccRCC cell lines

**Fig 1:**
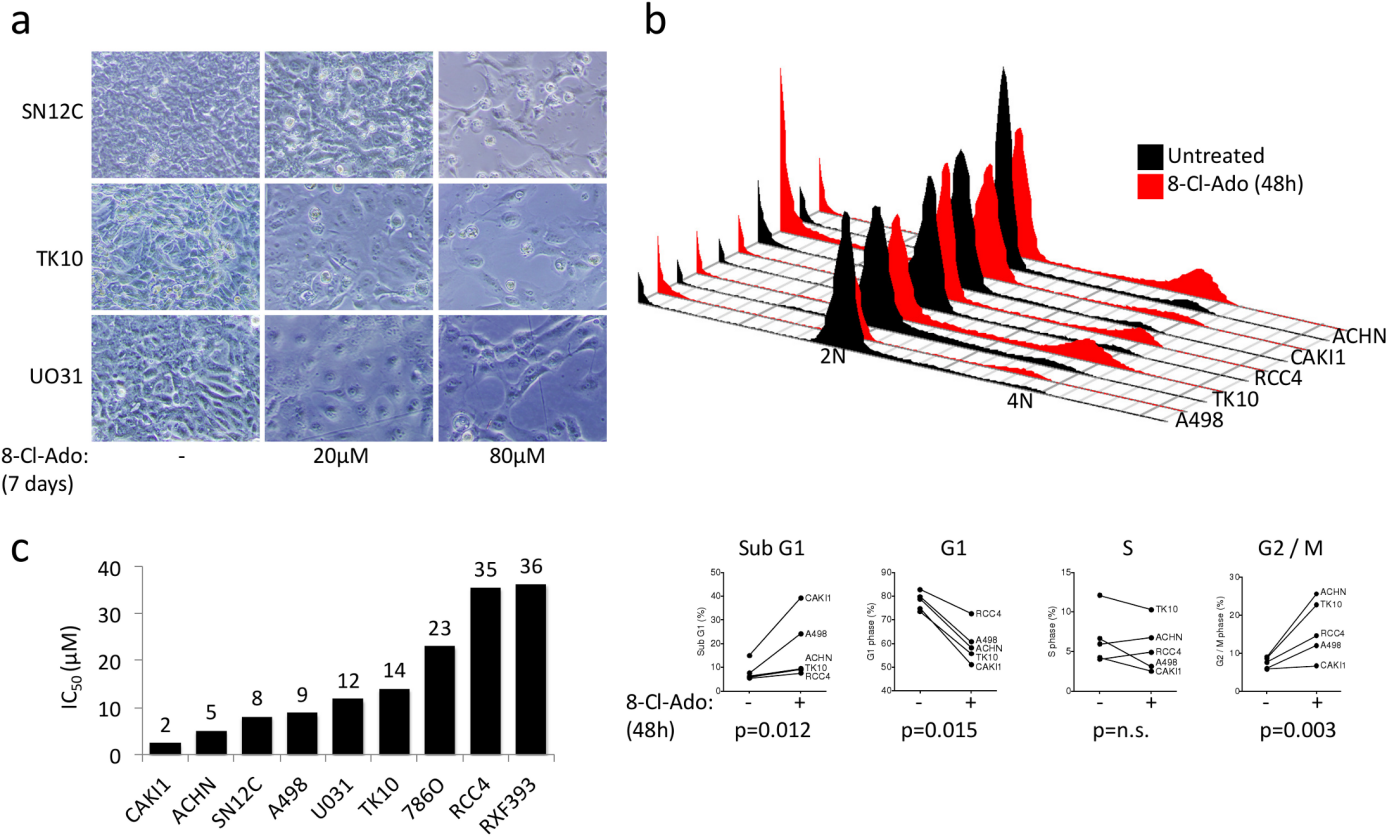
Efficacy of 8-chloroadenosine in ccRCC cell lines. **a.** ccRCC cell lines treated with 20 μM or 80 μM 8-chloroadenosine for 7 days. **b.** Cell cycle analysis of ccRCC cell lines treated with 40 μM 8-chloroadenosine for 48 hours. **c.** IC_50_ values of ccRCC cell lines calculated in Calcusyn software.

To our knowledge, 8-chloroadenosine has not been previously tested in ccRCC cell lines. First, in a pilot experiment, we determined that 8-chloroadenosine treatment leads to reduced proliferation and/or cell death by 7 days in SN12C, TK10, and UO31 cells (Fig 1A). We also observed an increase in G2/M phase of the cell cycle in most cell lines after 48 hours of treatment and an increase in cell death in the most sensitive cell line, CAKI-1 (Fig 1B). Next, we quantified the in vitro efficacy of 8-chloroadenosine in nine ccRCC cell lines (Fig 1C). We found the IC_50_ values to range from 2 μM to 36 μM. CAKI-1 and ACHN cell lines were the most sensitive, while RCC4 and RXF-393 were the most resistant.

### AMPK is activated upon 8-chloroadenosine treatment

**Fig 2:**
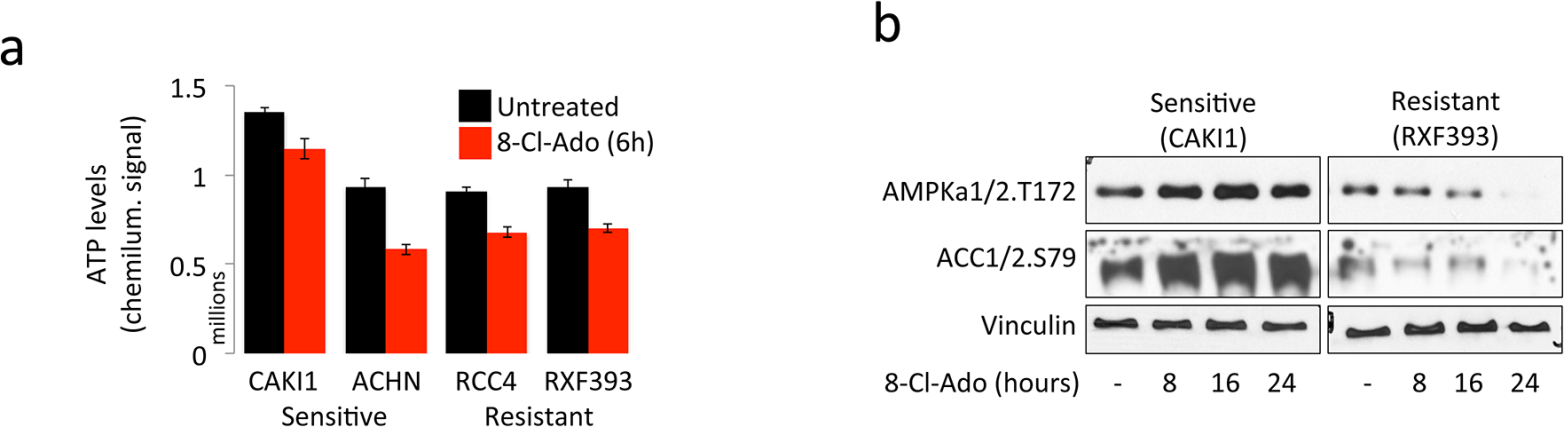
Decrease in ATP and AMPK activation in sensitive and resistant cell lines. **a.** ATP levels in sensitive and resistant ccRCC cell lines after 6 hours of 40 μM 8-chloroadenosine treatment. ATP levels decreased significantly in all cell lines (CAKI1 p<0.01, ACHN p = 0.02, RCC4 p<0.01, RXF393 p<0.01); however, ATP levels were not significantly different between the sensitive and resistant cell lines (p = n.s.). **b.** Western blots for phospho AMPK T172 and AMPK activity readout phospho ACC S79 after 0, 8, 16, and 24 hours of treatment with 40 μM 8-chloroadenosine. Vinculin is used as loading control.

8-chloroadenosine treatment has been reported to decrease ATP in cells by converting ATP to 8-chloro-ATP via ATP synthase [13]. Hence, we postulated that resistant cell lines may have higher pre-treatment ATP levels or may have less of a decrease in ATP levels after 8-chloroadenosine treatment. In all cell lines, ATP levels decreased (CAKI p<0.01, ACHN p = 0.02, RCC4 p<0.01, and RXF393 p<0.01). However, we found comparable levels of ATP before and after treatment in sensitive and resistant cell lines (p = 0.65, Fig 2A).

ATP depletion is known to activate AMP-activated kinase (AMPK), which gauges the AMP and ATP levels of the cell and orchestrates responses to energy crises. AMPK is activated when its threonine 172 residue (T172) in the alpha catalytic subunit is phosphorylated [19]. T172 phosphorylation of the alpha subunit requires AMP binding on the gamma regulatory subunit, which exposes the T172 site. Of relevance, 8-chloroadenosine has been recently reported to activate AMPK in breast cancer cells [9]. To find out whether 8-chloroadenosine treatment activates AMPK in ccRCC as well, we treated the most sensitive (CAKI-1 and ACHN) and the most resistant (RCC4 and RXF-393) cell lines for 8, 16, and 24 hours. Indeed, there was an increase in AMPK T172 phosphorylation, but only in the sensitive cells (Fig 2B). AMPK activity, measured by phosphorylation of acetyl-coA carboxylase (ACC) on residue serine 79 by AMPK, also increased in the sensitive cell lines. Therefore, 8-chloroadenosine increases AMPK phosphorylation and activity in sensitive ccRCC cell lines.

### mTOR pathway is inhibited upon 8-chloroadenosine treatment

**Fig 3:**
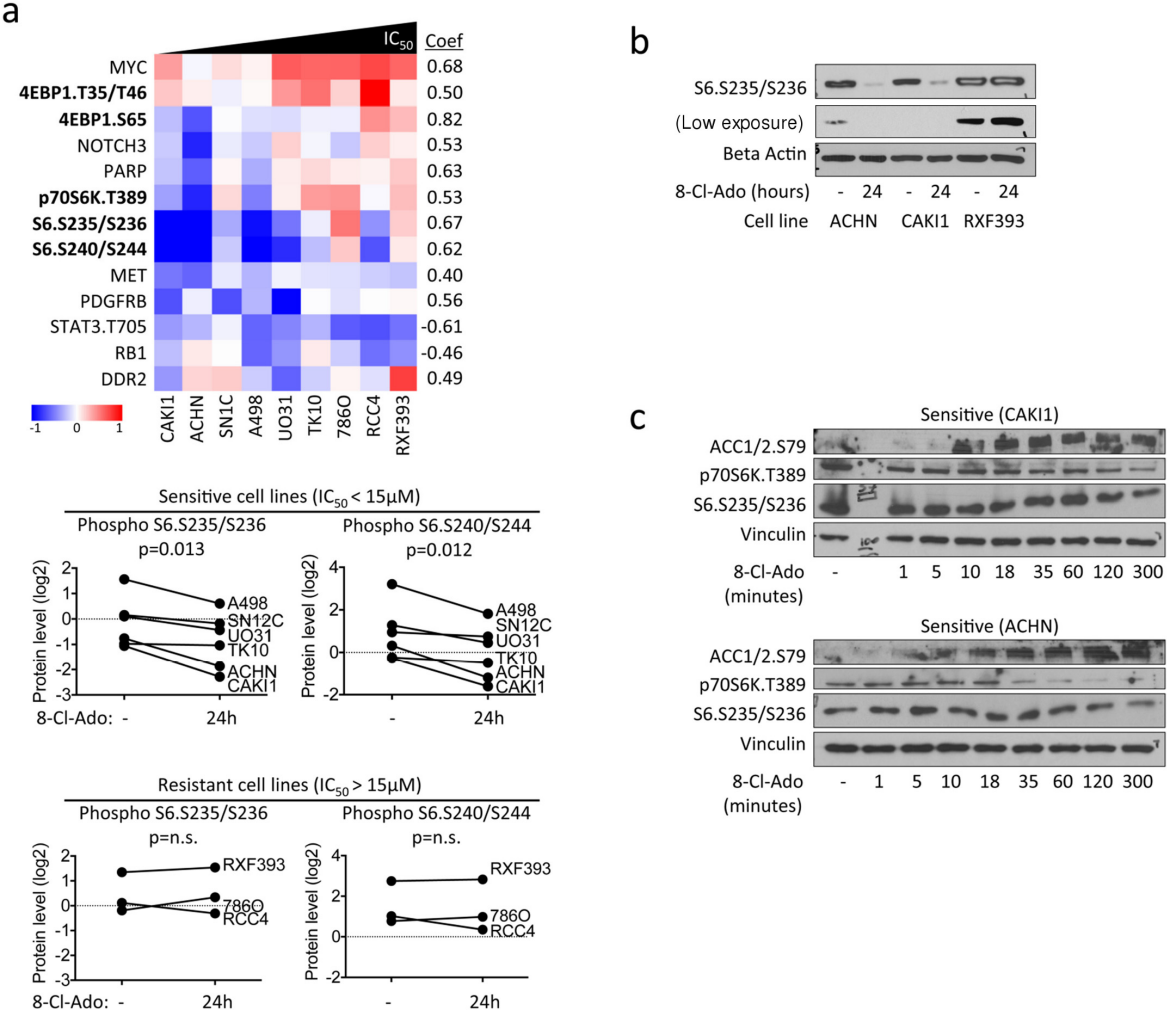
mTOR pathway downregulation in sensitive cell lines upon treatment with 8-chloroadenosine. **a**. RPPA heatmap showing expression changes that correlate with IC_50_ values. ccRCC cells were treated with 40 μM 8-chloroadenosine for 24 hours. Graphs show changes in phospho S6 levels in sensitive and resistant cell lines. p-values are calculated using two-tailed paired t-test. coef. Pearson correlation coefficient between the IC_50_ values and levels of proteins measured by RPPA. **b**. Western blot validating phospho S6 S235/236 downregulation in CAKI1 and ACHN cell lines upon 8-chloroadenosine treatment for 24 hours. **c**. Western blot showing phospho ACC S79 as readout of AMPK activity and phospho p70S6K T389 and phospho S6 S235/236 for mTOR pathway activity. ccRCC cells were treated with 40 μM 8-chloroadenosine for 1, 5, 10, 18, 35, 60, 120, or 300 minutes. Vinculin served as loading control.

To investigate the signaling pathways associated with the differential sensitivity of ccRCC cell lines to 8-chloroadenosine, we performed reverse-phase protein arrays (RPPA). We analyzed levels of total and phosphorylated proteins in various signaling pathways, including MAPK, PI3K, and mTOR. The protein changes that correlated with the IC_50_ values included decrease in phospho S6.S235/236 and phospho 4EBP1.S65 in the sensitive, but not resistant, cell lines, and increase in MYC in the resistant cell lines (Fig 3A). We validated S6 dephosphorylation at S235/236 by western blot (Fig 3B).

AMPK, as the energy sensor of the cell, can shut down energy expending processes, such as protein and lipid syntheses. AMPK decreases synthesis of acetyl-coA into fatty acids by inhibiting the enzyme acetyl-coA carboxylase (ACC) through phosphorylation of residue S79. AMPK activation also decreases protein synthesis by phosphorylating TSC1/2 TOR and resulting in mTOR pathway inhibition. To establish whether AMPK activation by 8-chloroadenosine precedes mTOR pathway inhibition, we performed a time-course experiment. We found rapid increase in AMPK activity, measured by ACC phosphorylation, within the first 10 minutes of 8-chloroadenosine treatment, followed by mTOR pathway inhibition, measured by p70 S6K dephosphorylation, between 30 minutes and 2 hours (Fig 3C). Finally, S6 dephosphorylation took place after 2 hours. Our observations support rapid activation of AMPK followed by shutdown of the mTOR pathway in response to 8-chloroadenosine treatment.

### Resistance to 8-chloroadenosine is associated with PI3K pathway activity

**Fig 4:**
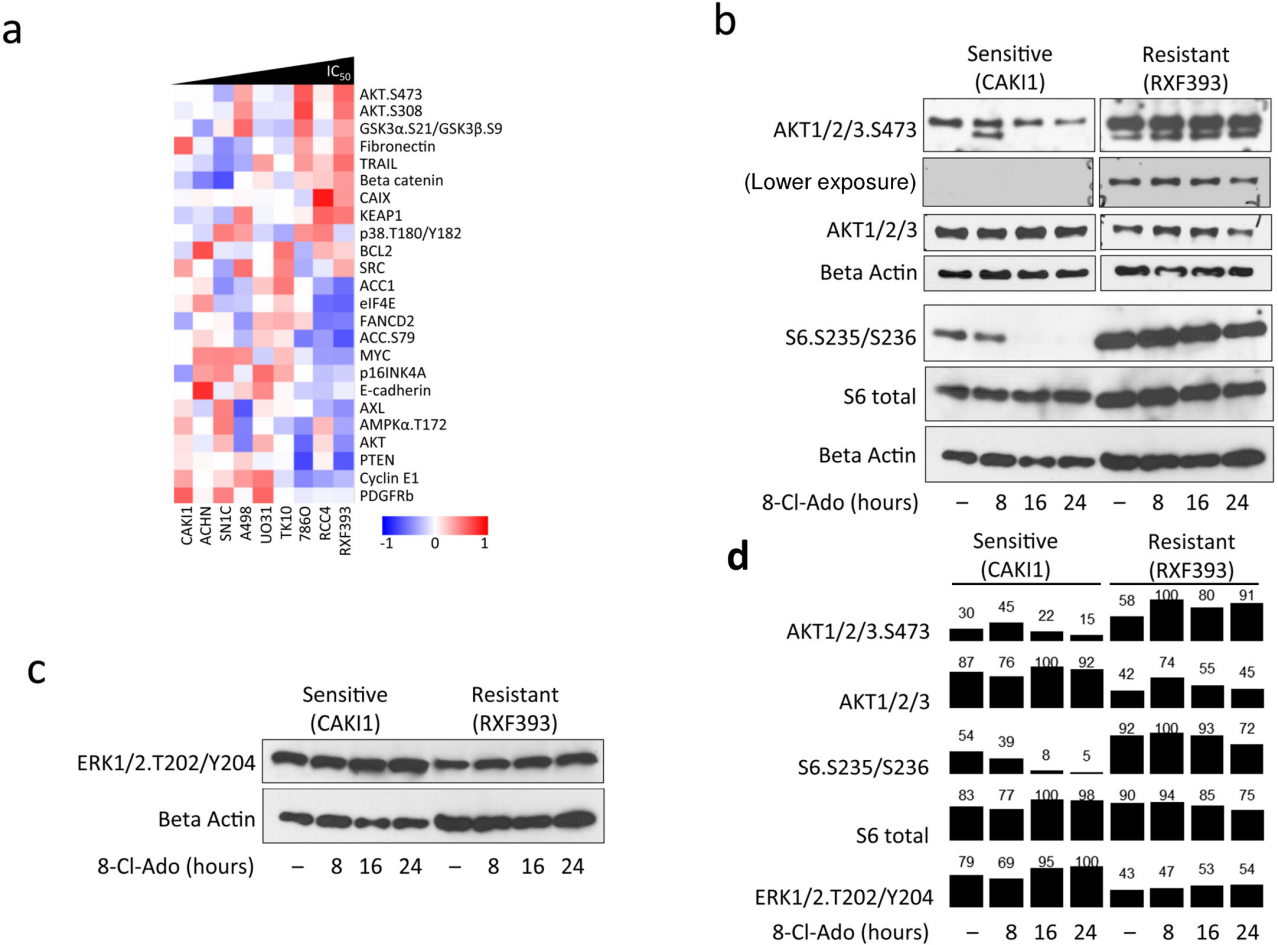
PI3K pathway is active at baseline in 8-chloroadenosine resistant, but not in sensitive, cell lines. **a**. RPPA heatmap showing proteins that are differentially expressed between the most sensitive and resistant cell lines at baseline prior to treatment. AKT phospho S473 and S308 were higher and phospho AMPK T172, ACC S79, and total PTEN were lower in the resistant cell lines. **b**. Western blots showing phospho and total AKT and S6 levels in sensitive and resistant cells before and after 40 μM 8-chloroadenosine treatment. Beta-actin served as loading control. **c**. Western blots showing phospho ERK T202/204 levels in sensitive and resistant cells before and after 40 μM 8-chloroadenosine treatment. Beta-actin served as loading control. **d**. Quantification of phospho AKT, S6, and ERK and total AKT and S6 levels.

The role of the PI3K pathway in mTOR pathway activation and resistance to mTOR inhibition is well established. Indeed, through RPPA, we found greater baseline levels of phospho AKT S473 and phospho AKT T308 in the resistant cell lines (Fig 4A). Using western blot, we validated phospho AKT S473 levels in the most sensitive and the most resistant cell lines, CAKI-1 and RXF-393, respectively (Fig 4B, quantified in Fig 4D).

Resistant cell lines, where AKT is constitutively active, avoid AMPK phosphorylation at T172 (Figs 2B, 4A and 4B). A recent study showed that AKT can phosphorylate AMPK at T487, and as a result preclude AMPK phosphorylation at T172, blocking AMPK activation [20]. We speculate that in the cells lines that are resistant to 8-chloroadenosine, constitutively active AKT precludes AMPK activation. Therefore, AKT may block effects of 8-chloroadenosine through direct activation of mTOR pathway and/or through inhibition of AMPK.

Another pathway known to regulate mTOR activity is the MAPK pathway. Hence, we wondered whether the resistant cell lines would also have higher levels of MAPK activity. However, we did not find a consistent difference in phospho ERK T202/204 levels before treatment between the sensitive and resistant cell lines by RPPA and Western blot (Fig 4C, quantified in Fig 4D). These results suggest that sensitivity to 8-chloroadenosine in ccRCC cell lines is associated with an activated PI3K pathway.

### PI3K pathway inhibition improves efficacy of 8-chloroadenosine

**Fig 5:**
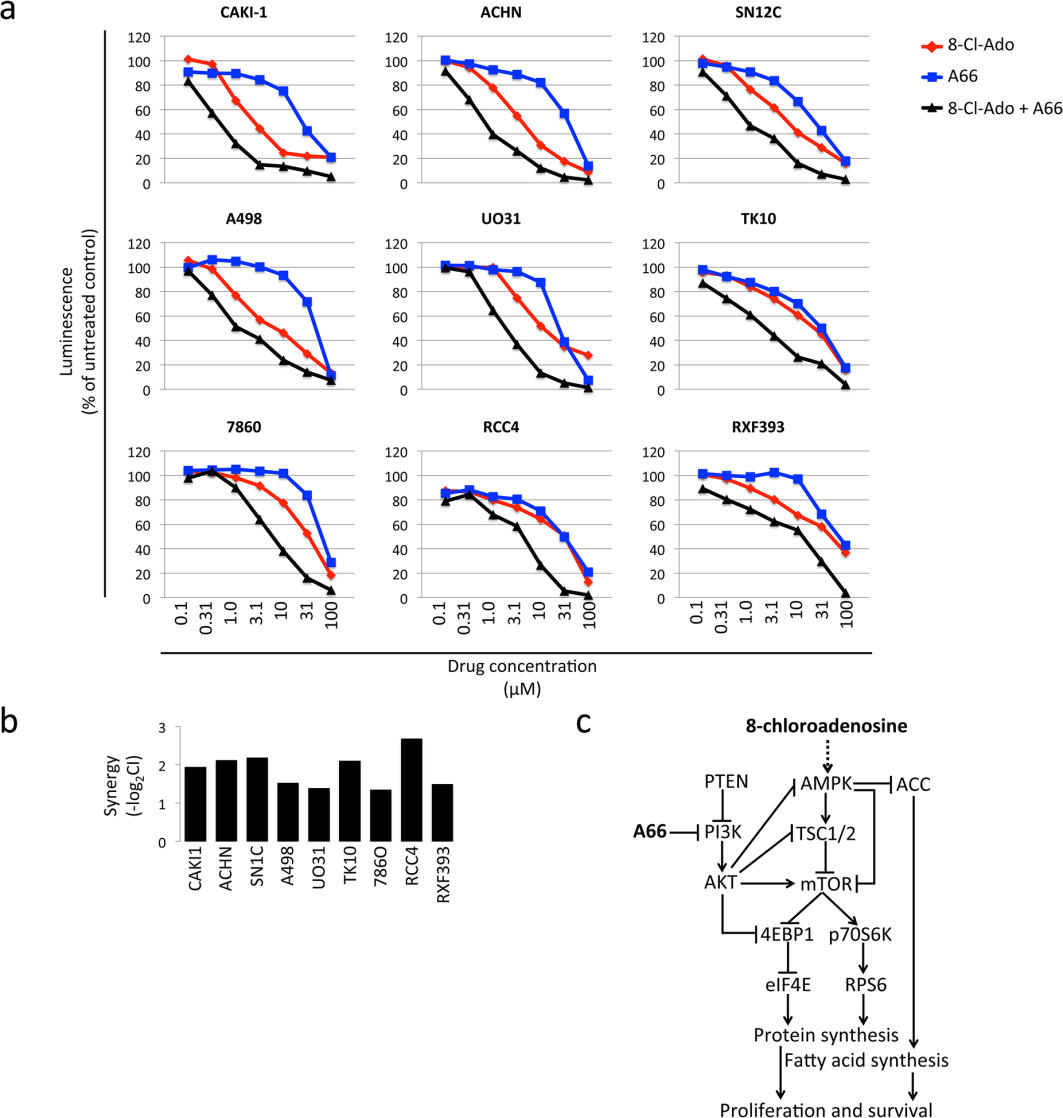
8-chloroadenosine synergizes with PI3K inhibitor A66. **a.** Cell proliferation measured by chemiluminescence after treatment with 8-chloroadenosine, PI3K inhibitor A66, or the combination. Cells were plated in 96-well plates and treated with 8-chloroadenosine, PI3K inhibitor A66, or a combination of the two at concentrations ranging from 0.1 μM to 100 μM. After 96 hours of treatment, proliferation was measured. Treatment with the combination of 8-chloroadenosine and A66 decreased proliferation more than treatment with either drug alone. **b**. Synergy index, defined as negative logarithm of CI (combination index) for 8-chloroadenosine and A66 across nine RCC cell lines. A positive score indicates synergy, a score of zero indicates additive effect, and a negative score indicates antagonism between the two drugs. Chou-Talalay method in the Calcusyn software was used to calculate the synergy between the two drugs. **c**. Model illustrating how 8-chloroadenosine may affect RCC cells through AMPK and mTOR pathway.

To determine whether activated PI3K contributes to 8-chloroadenosine resistance, we inhibited the PI3K pathway using the PI3K p110α inhibitor A66. We found the combined treatment more effective than either 8-chloroadenosine or A66 alone (Fig 5A). Moreover, there was synergy between the two drugs in each of the nine ccRCC cell lines tested (Fig 5B). We conclude that an active PI3K pathway contributes to resistance to 8-chloroadenosine.

## Discussion

The adenosine nucleoside analog 8-chloroadenosine is a novel drug candidate with inhibitory activity against malignant hematological and epithelial cells. 8-chloroadenosine cytotoxicity is thought to be mediated through ATP depletion, but there is no consensus on the molecular pathways critical to its effects. In ccRCC cell lines, we found that 8-chloroadenosine is cytotoxic in vitro, and sensitivity to 8-chloroadenosine is associated with AMPK activation and mTOR pathway inhibition. Moreover, cells with constitutive PI3K pathway activation were more resistant to 8-chloroadenosine and pharmacological inhibition of PI3K pathway synergized with 8-chloroadenosine treatment.

8-chloroadenosine demonstrated an inhibitory effect in ccRCC cells, with IC_50_ values ranging from 2 μM to 36 μM (Fig 1). In the more sensitive cells, 8-chloroadenosine led to rapid AMPK activation and subsequent inhibition of the mTOR pathway (Fig 3). The cells resistant to 8-chloroadenosine were characterized by lower AMPK and higher PI3K pathway activity at baseline before treatment (Fig 4), thus favoring sustained mTOR pathway activity. We noted lower baseline levels of AMPK phospho T172 and lack of further AMPK phosphorylation upon treatment in the resistant cell lines. A previous study revealed that downregulation of AMPK activity by AKT; this seems to be the case in the ccRCC cell lines that are resistant to 8-chloroadenosine [20]. Either through precluding AMPK activation or mTOR inhibition, activated PI3K pathway plays a key role in conferring resistance to 8-chloroadenosine. Concordantly, pharmacological inhibition of PI3K synergized with 8-chloroadenosine treatment (Fig 5).

AMPK is a major regulator of the mTOR pathway. Our results suggest, but not prove, that 8-chloroadenosine’s inhibitory effect on mTOR pathway may be through the activation of AMPK. As the energy sensor of the cell, AMPK is a key decision-maker about whether a cell can afford the resources to proliferate. When energy levels in the cell are low, AMPK downregulates synthesis of proteins and lipids that are necessary building blocks of cells. To downregulate protein synthesis, AMPK phosphorylates TSC2, which in turn inhibits mTOR pathway, leading to dephosphorylation of ribosomal subunit component RPS6 (S6) and sequestration of translation initiation factor 4E (eIF4E). To downregulate lipid synthesis, AMPK phosphorylates and inactivates ACC, halting production of malonyl-CoA for fatty acid biosynthesis. We think that 8-chloroadenosine, through AMPK activation, tricks cells into an energy-saving starvation mode, and consequently results in the shutdown of protein and fatty acid syntheses, jeopardizing proliferation and survival of cells (Fig 5C). In contrast, AKT in the PI3K pathway opposes AMPK’s inhibitory effects on the mTOR pathway, both through inhibition of AMPK and TSC2 and direct activation of mTOR itself (Fig 5C). Our finding of high phospho AKT and a lack of AMPK activation and mTOR pathway inhibition in resistant cell lines is consistent with PI3K pathway antagonizing the effects of 8-chloroadenosine. Concordantly, pharmacological inhibition of PI3K by A66 synergized with 8-chloroadenosine treatment in inhibiting proliferation of ccRCC cell lines.

AMPK activation may have therapeutic potential. However, despite promising results from AMPK activation studies in vitro, it is not clear whether AMPK activation will have anti-cancer efficacy [21, 22]. AMPK has been shown to have both pro- and anti-tumorigenic effects. On one hand AMPK helps cancer cells survive under stress conditions; on the other hand, AMPK activation slows down protein synthesis and proliferation.[23] Specifically in ccRCC, AMPK activation has been suggested to have anti-tumorigenic effects in vitro and in vivo [24–26]. We surmise efficacy of AMPK activation against cancer may depend on type and extent of energy stress as well as the tools the cancer cell has at its disposal for survival. While dependence on mTOR pathway may pose vulnerability, the ability to activate PI3K in response to energy stress may confer resistance.

Further evidence for anti-tumorigenic effect of AMPK activation in ccRCC comes from The Cancer Genome Atlas (TCGA) ccRCC patient data set (KIRC), which revealed that functional AMPK (phosphorylated at T172) is associated with prolonged survival [27]. It is therefore possible activating AMPK may have therapeutic potential in ccRCC patients. ccRCC is often responsive to mTOR inhibitors, and AMPK activation may be an effective strategy to therapeutically exploit this vulnerability. Moreover, AMPK activation also brings to the table additional benefits, such as inhibition of fatty acid synthesis, induction of autophagy, and a probable shutdown of a broader swath of pro-tumorigenic cellular processes [28–31].

Challenges remain in moving 8-chloroadenosine to the oncology clinic despite the observed activity. In mice, the half-life of parenteral 8-chloroadenosine is approximately one hour, and attempts at improving its pharmacokinetic properties have achieved limited success [32]. On the promising side, clearance of the intracellular 8-chloro-ATP and other metabolites of 8-chloroadenosine is slow [33]. In due course, the efficacy of 8-choroadenosine on solid tumors needs to be tested in vivo, probably after chemical modification or optimization of formulation or delivery.

Emerging ATP depletion therapies, such as 8-chloroadenosine, have potential to provide a new class of cancer drugs. Energy starvation of cancer cells not only downregulates the mTOR pathway, but also likely inhibits fatty acid synthesis and perturbs redox balance, leading to broad changes in cellular metabolism. Here we have described how 8-chloroadenosine activates AMPK and subsequently inhibits the mTOR pathway in sensitive RCC cells. We found that resistance to 8-chloroadenosine is associated with an activated PI3K pathway. Because of the importance of the mTOR pathway in ccRCC and the role AMPK, the energy sensor of the cell, plays in the regulation of the mTOR pathway, ccRCC may be an appropriate model for developing ATP depletion therapies. Ultimately, we believe our results will give impetus to future studies developing ATP depletion therapies in ccRCC.
